# Quantification of Volatile Compounds in Mixtures Using a Single Thermally Modulated MOS Gas Sensor with PCA–ANN Data Processing

**DOI:** 10.3390/s25226913

**Published:** 2025-11-12

**Authors:** Jolanta Wawrzyniak

**Affiliations:** Faculty of Food Science and Nutrition, Poznań University of Life Sciences, 60-624 Poznań, Poland; jolanta.wawrzyniak@up.poznan.pl

**Keywords:** thermally modulated metal-oxide semiconductor gas sensor, MOS gas sensor, artificial neural network (ANN), machine learning, principal component analysis (PCA), ethanol and methanol mixture, volatile compound quantification

## Abstract

**Highlights:**

**What are the main findings?**

- Methodology enhancing the selectivity of MOS gas sensors for measuring ethanol and methanol levels in liquid mixtures.
- Approach to interpreting the output signals of thermally modulated MOS gas sensor by integrating two data processing techniques: PCA and ANN.

**What is the implication of the main finding?**

- The proposed methodology, integrating measurements from a single thermally modulated MOS gas sensor with a PCA–ANN-based machine learning algorithm, can be applied for the quantitative determination of VOCs in mixtures and demonstrates potential applicability to other types of sensors and volatile compounds.

**Abstract:**

Recent research efforts have focused on improving the performance of metal-oxide semiconductor (MOS) gas sensors through their thermal modulation using integrated heaters. This approach allows us to enhance the selectivity of measurements; however, the main challenge with this amelioration lies in interpreting the sensor response, which takes the form of complex patterns that require the application of advanced signal processing techniques. This study introduces a methodology for the quantitative determination of volatile compounds (ethanol and methanol at various concentrations ranging from 31 to 2000 ppm for each of these compounds) in mixtures using a single thermally modulated MOS gas sensor. The recorded responses of the detector were interpreted by combining two signal processing techniques: principal component analysis (PCA) for feature extraction, and artificial neural networks (ANNs) for predicting the levels of the tested volatile components. The proposed methodology demonstrated satisfactory performance achieving R^2^ values at the level of 0.999 across all datasets (learning, test, validation) and low error metrics (RMSE = 11.6–14.4 ppm), thereby confirming the robustness and accuracy of the approach and its applicability in a wide range of fields where rapid, cost-effective, and precise detection of ethanol and methanol is essential.

## 1. Introduction

Rapid and reliable analysis of ethanol and methanol mixtures with the potential for automation is crucial due to their widespread industrial applications. Of the two alcohols, ethanol is commonly used in food, pharmaceuticals, and biological applications, while methanol is utilized in chemical and petrochemical industries for dyeing, antifreeze, biodiesel, petroleum, and chemicals production [1,2]. In many cases, for example, in the production of industrial alcohol and disinfectants, methanol is often added to ethanol to reduce costs. Although both of these volatile organic compounds (VOCs) share similar chemical and physical properties, they differ significantly in their toxicity. Ethanol, a common component of many products encountered in daily life, can cause a range of health problems, from mild irritation of the respiratory and digestive tracts, through cardiovascular disorders and neurological impairments, to severe liver and kidney damage, and even cancer, when consumed in large amounts or over a long period [3,4]. It should be noted that while a single episode of heavy ethanol consumption (particularly among young people) can lead to severe poisoning or even death, the majority of ethanol-related health problems arise from its prolonged consumption, even at moderate levels. Methanol, on the other hand, is not consumable, and even when inhaled, absorbed through the skin, or ingested once, even in very small doses can cause permanent damage to the nervous system and eyes, and even death [5]. Considering similar properties and the fact that both alcohols can be used interchangeably in many applications, yet differ greatly in their toxicity, special attention should be devoted to developing effective methods and more advanced techniques for detecting ethanol and methanol. Among these, gas chromatography-mass spectrometry (GC-MS) [6] and nuclear magnetic resonance (NMR) [7] are widely regarded as the gold standard for VOC identification. However, their practical application in daily life is restricted by high equipment costs and the necessity for specialized expertise. In this context, gas sensing technologies based on different type of gas sensors (electrochemical [8], fluorescent [9], chemoresistive [10]) have emerged as a promising alternative solution due to their cost-effectiveness, simplicity, high sensitivity, and potential for application in real-time monitoring. Among them, particular attention should be given to chemoresistive (metal-oxide-semiconductor (MOS)) sensors, whose operating principle is based on changes in the electrical resistance of their metal-oxide sensing layer, caused by interactions with target gases. This makes them suitable for numerous applications across various fields, including air quality monitoring, industrial leak detection, alarm systems, food quality control, automotive emissions regulation, and in medicine diagnostic tools [11,12,13,14,15,16,17,18,19]. Nevertheless, despite notable achievements, both the sensors themselves and the systems in which they operate still require further improvements in terms of enhanced selectivity, faster response and recovery times, reduced dependence on humidity and temperature, and ultra-low detection limits. The use of MOS gas sensors is particularly constrained by their low selectivity, as a single sensor exposed to a mixture of volatile compounds produces an output signal that represents the combined response to all present substances, making it difficult to distinguish their individual contributions [20,21]. This cross-sensitivity leads to poor selectivity, especially for compounds with similar physicochemical characteristics (gas analogs), such as ketones and alcohols, which elicit comparable signal intensities from the sensor [21,22,23]. Consequently, quantitative analysis of volatile compounds with single MOS gas sensors operating within a conventional setup (ensuring constant sensor operating temperature) is only reliable in systems containing a single, well-defined type of compound.

Previous reviews widely discuss the advancements made in MOS-based gas sensor development, including material selection, fabrication techniques, and performance optimization strategies aimed at improving MOS gas sensor performance and expanding their application [24,25,26,27]. Numerous studies focused on enhancing the sensitivity, selectivity, and stability of MOS gas sensors have focused on the development of novel or modified sensing materials, including nanostructuring, metal nanoparticle incorporation, and elemental doping [10,28,29,30,31,32,33,34]. Another promising approach to improving selectivity involves the construction of matrices consisting of several sensors, the response of which generates the summary response signal carrying a more information about nature of the tested mixture of gas components [35,36,37], or the use of thermal modulation using a heater integrated with MOS gas sensors [38,39,40]. The application of the latter technique means that the behavior and responses of a single sensor are comparable to those of a sensor array composed of several sensors of the same type, each operating at different temperatures. A thermally modulated sensor allows for the collection of more comprehensive information about the analyzed sample; however, the challenge with this approach lies in interpreting the sensor response, as it generates a complex pattern that requires the application of advanced signal processing techniques. In previous studies, decoding sensor response patterns was commonly conducted with use of principal component analysis (PCA) [39,41,42] and partial least squares (PLS) regression [35]. As it was found that applying statistical shape space pre-processing to temperature-modulated signals from metal oxide sensors significantly improved gas identification [43], sometimes, PCA or linear discriminant analysis (LDA) were combined with other machine learning techniques like k-nearest neighbor (KNN), logistic regression (LR), support vector machine (SVM), or random forest (RF) algorithms [44,45,46]. Recent studies have shown promising results using advanced regression techniques based on B-splines combined with artificial neural networks, as well as deep learning–based convolutional neural networks (CNNs), to interpret waveforms generated by gas sensor array or a single thermally modulated MOS gas sensor for quantifying volatile components in mixtures [21,40,47]. Building on these findings and aiming to further expand the application potential of thermally modulated MOS gas sensors, the present research explores an alternative approach by developing a methodology that integrates traditional signal processing techniques, such as PCA, with an artificial neural network (ANN)-based machine learning algorithm, enabling a rapid and relatively simple determination of volatile compound concentrations (ethanol and methanol) in mixtures using a single thermally modulated MOS gas sensor.

## 2. Materials and Methods

### 2.1. Measurement System and Methodology

The detection of volatile compounds was carried out using a single TGS2610-C metal oxide semiconductor (MOS) gas sensor, produced by Figaro Engineering Inc., Osaka, Japan. This sensor is built on the basis of an alumina substrate covered with the gas-sensitive component in the form of thin tin oxide (SnO_2_) semiconductor layer that is integrated with a micro-heater consisting of a printed ruthenium (RuO_2_) layer. When the sensor connected to the measuring system is exposed to the tested atmosphere, an equilibrium is established between the amount of substances adsorbed on the sensor surface and their concentration in the surrounding atmosphere. The adsorbed substances may undergo redox reactions (especially at elevated temperatures resulting from the operation of the micro-heater) and exchange electrons with each other and with the sensor surface. The processes described above lead to changes in the number of charge carriers within the sensor layer, which are manifested as variations in the sensor resistance, changes which can be interpreted as information about the concentration of the analyzed compounds.

**Figure 1:**
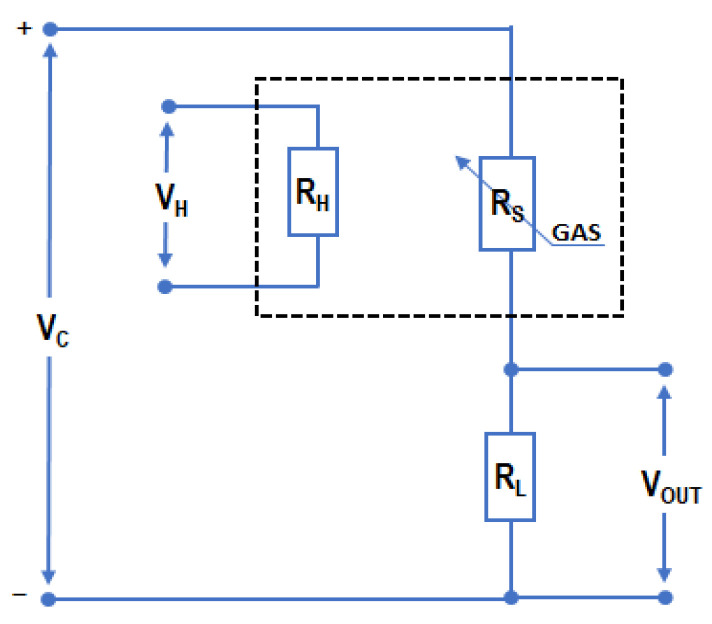
Schematic diagram of the measurement system for the TGS2610-C sensor. R_S_ represents the sensor resistance, R_L_ denotes the auxiliary (load) resistor, while V_C_ and V_H_ correspond to the voltages applied to the sensor circuit and micro-heater circuit, respectively. V_OUT_ is the output voltage measured across a voltage divider that incorporates the auxiliary resistor R_L_, providing a signal related to the sensor resistance R_S_ [21].

A typical measurement circuit for this sensor works as a voltage divider, where variations in the sensor resistance (R_S_) are monitored indirectly by measuring the output voltage signal (V_OUT_) at an auxiliary resistor, as illustrated in Figure 1. According to the manufacturer standard operating procedure, the sensor (V_C_) and the micro-heater (V_H_) should be supplied with a constant voltage of 5.0 V. To improve the sensor selectivity, in the study the conventional approach was modified by incorporating thermal modulation of the sensor by powering its micro-heater with varying voltage. Modulating the sensor temperature allows us to regulate the rate of the adsorption and redox reaction of the target substances on the sensor surface, thereby enhancing the dynamics of sensor resistance changes and providing additional analytical information about the sample.

Previous research indicates that applying various modulation patterns to the heater, including ramp, rectangular, sinusoidal, saw-tooth, sigmoid, triangular ones allows for a distinction sensor response signatures corresponding to specific analytes [42,44,45,48]. Some of those investigations have demonstrated that ramp, saw-tooth, and triangular voltage waveforms offer strong discrimination capabilities for detecting VOCs [21,39,40,43,49]; therefore, based on these findings, a simple saw-tooth waveform was also selected to regulate the micro-heater voltage (V_H_) in the presented research.

**Figure 2:**
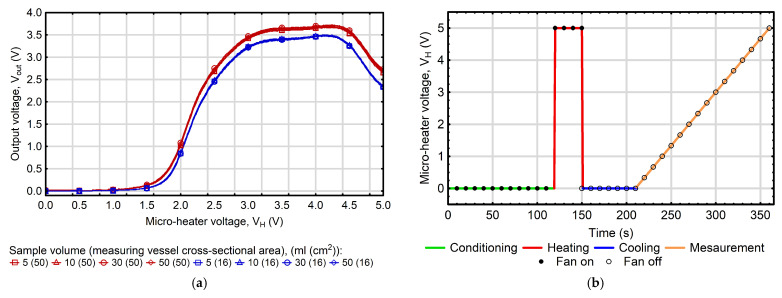
(**a**) Effect of sample volume and cross-sectional area of the measurement vessel on the responses of the thermally modulated MOS gas sensor. (**b**) The dynamics of the voltage supply to the micro-heater of a thermally modulated MOS gas sensor during the quantitative detection of volatile compounds in liquid mixtures.

In the study, the analysis of selected volatile compounds was conducted in a 2 L chamber equipped with the above-described thermally modulated MOS gas sensor and a small fan to ensure atmosphere homogenization. Tested samples were aqueous solutions containing known concentrations of ethanol and methanol. Each measurement involved introducing 50 mL of the prepared earlier solution into the chamber, nevertheless, the preliminary experiments, in which additional analyses were performed using samples of 5, 10, 20, 30, 40, and 50 mL, showed that the proposed approach remained effective even for samples with volumes as small as 5 mL and that the sensor response was influenced to a much greater extent by the surface area of the sample exposed to the chamber atmosphere than by the total sample volume (Figure 2a). The single measurement cycle lasted 360 s and began with a 120 s period (with the fan turned on) intended to establish thermodynamic equilibrium between the liquid and gas phases. In the subsequent step, with the fan still operating, the micro-heater was supplied with a voltage of 5 V for 30 s. This step was aimed at conditioning the sensor by removing or reacting substances adsorbed on its surface during previous measurements. Afterwards, the fan and heater power were turned off, and the sensor was allowed to cool for 60 s. Following these preparatory steps, the main measurement was initiated, during which the heater was supplied with a linearly increasing voltage from 0 to 5 V at a rate of 2 V/min (over 150 s). During this period, the response patterns of the MOS gas sensor (which contained information related to the composition of tested samples) were recorded as the relationship between the micro-heater voltage (V_H_) and the output signal voltage (V_OUT_). Figure 2b depicts a graph showing the dynamics of voltage supply to the micro-heater of a thermally modulated MOS gas sensor. To ensure measurement reliability, each test was performed in triplicate. Prior to each measurement, the chamber was ventilated with fresh air for 5 min to eliminate residual gases and prevent cross-contamination.

The experiments were conducted under controlled environmental conditions, maintaining a temperature of 22–24 °C and a relative humidity (outside the measuring chamber) of 35–45%. The RH can potentially act as an interfering factor in gas sensor measurements, and its influence should be considered when evaluating the applicability of the sensor in various environments; however, in the present study RH remained stable, as all measurements were carried out in an atmosphere that was in equilibrium with the aqueous solutions of the analyzed substances achieved after a stabilization period following sample introduction into the measurement chamber, as presented in one recent study [40]. The optimal operating parameters of a measurement system working with a linearly increasing heater voltage, which allow the measurement cycle to be maintained at reasonable duration and produce well-defined output patterns, were estimated in device test described in a previous study [40]. Its results demonstrated that the measurement system reached stability within less than 60 s after the sensor was powered on (corresponding to approximately 90 s after the sample was introduced into the measurement chamber). The findings also revealed that a 30 s interval was adequate for the micro-heater to cool down completely, as indicated by signal stabilization following power deactivation.

### 2.2. Preparation of Analyzed Solutions

The research investigated aqueous mixtures based on deionized water containing two volatile organic compounds: ethanol and methanol. Ethanol with a minimum purity of 99.5% and methanol with a purity of at least 99.8% were sourced from Merck. The study involved 13 different concentrations of each compound, ranging from 31 to 2000 ppm. Ethanol–methanol mixtures were prepared using a serial dilution method including all possible combinations of the following concentrations: 2000, 1414, 1000, 707, 500, 354, 250, 177, 125, 88, 63, 44, and 31 ppm, leading to a total of 169 distinct measurement samples.

### 2.3. MOS Gas Sensor Output Signals Processing

The average response profiles (waveforms) of the thermally modulated gas sensor, created from three measurement cycles per sample recorded during the experiments, encode information about the composition of the solution. Therefore, they may serve as suitable input signals for estimating ethanol and methanol concentrations in liquid mixtures. As each recorded waveform, illustrating the relationship between the micro-heater voltage (V_H_) and the sensor output voltage (V_OUT_) consisted of 501 points (obtained over a V_H_ range 0 to 5 V with a resolution of 0.01), using it directly as an input vector for the model (e.g., an ANN model) could lead to a substantial increase in computational complexity. Therefore, it was necessary to reduce its dimensionality while preserving information on the composition of the analyzed solutions.

#### 2.3.1. Output Signal Feature Extraction

Principal component analysis (PCA) is one of the methods allowing to extract the relevant features and minimize dimensionality of the extended dataset. Considering its numerous advantages, in the study this method was applied to the original measurement results. During the analysis, redundancy among variables was reduced through the creation of principal components, which are new uncorrelated variables that capture the majority of the variance present in the original data. Once the input internally correlated variables were converted into a new set of independent components, the relevant number of principal components were determined by both Kaiser’s and Cattell’s criteria. The new dataset, reduced, but still retaining the essential features of the original dataset, could be used as input for model development without the fear of a rapid increase in computational complexity.

#### 2.3.2. Development of a Model for Estimating Liquid Mixture Composition

##### Data Preparation

To construct a neural network model for estimating the composition of liquid solutions containing tested volatile analytes (ANN_E-M_), the measurement cases, along with their associated input vectors (consisting of a set of principal components computed from all measurement points) and output vectors (comprising the analyte concentrations), were randomly divided into three subsets: learning (70%), test (15%), and validation (15%). Consequently, the ANN model was developed using data from 169 analyses, of which 119 cases were designated for learning, 25 for testing, and the remaining 25 for validation.

##### Artificial Neural Networks in Modeling of Liquid Mixtures Composition

Among feedforward neural networks, multilayer perceptrons (MLPs) and radial basis function networks (RBFs) are widely recognized for their effectiveness in capturing nonlinear phenomena commonly observed in the food industry and chemical engineering. In detail, this means that the signals fed to the input neurons are transmitted via weighted connections to the neurons of the hidden layer, where they are transformed by activation functions. Next, the signals processed in the hidden layer are transmitted through weighted connections to the neurons of the output layer, where they are transformed by the activation function to produce the final output signal.

**Figure 3:**
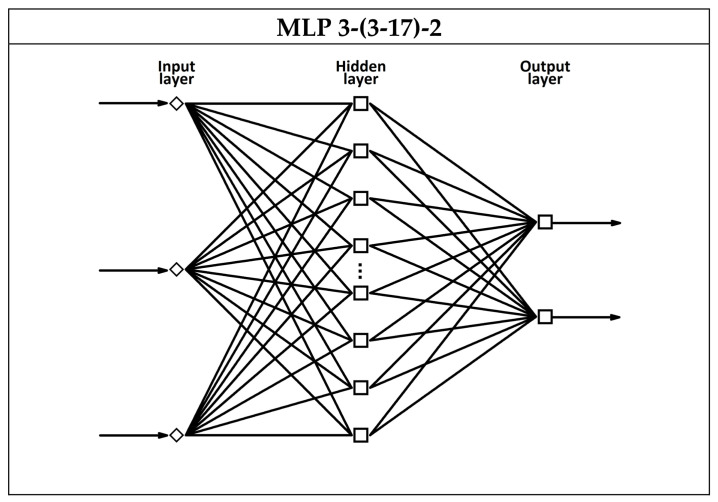
Diagram of the general structure of MLP neural networks employed to develop a model for estimating the composition of liquid mixtures containing ethanol and methanol, utilizing signals from a thermally modulated MOS gas sensor.

In the study, three-layer MLP structures were considered for modeling the composition of liquid mixtures containing ethanol and methanol, based on signals from a thermally modulated MOS gas sensor. The input layers consisted of neurons corresponding to independent variables, represented by identified principal components that encapsulated the most essential information from the original data, whilst the output layers comprised two neurons responsible for predicting the concentration levels of the studied analytes (ethanol and methanol). Optimization of the network architecture (general structure depicted in Figure 3) involved adjusting the size of the hidden layer by testing structures with three to seventeen neurons and evaluating various activation functions in the neurons of the hidden layer (logistic, hyperbolic tangent, exponential).

For each network architecture, one thousand models were generated, resulting in 45,000 MLP networks (15 hidden layer sizes × 3 types of activation functions × 1000 generated networks for each structure). Each simulation was performed independently, using a random split of the dataset into training, test, and validation subsets. The model learning process involved iterative adjustments of connection weights and activation function parameters to minimize learning error (E_l_). The root mean square error function served as the performance metric in the learning, test, and validation stage of model evaluation. The learning process was continued until either a predefined iteration limit was reached or an increase in test error (E_t_) indicated potential overfitting, prompting early termination. The MLP networks were learned using a Broyden–Fletcher–Goldfarb–Shanno (BFGS) algorithm suitable for nonlinear problems. During the optimization process, successful network topologies were taken to be those capable of making accurate predictions on new data that did not participate in the model development process, as assessed by the computation of validation error (E_v_). The structure with the lowest weighted average of learning, test, and validation errors was adopted as a model for estimating the composition of liquid mixtures containing ethanol and methanol.

### 2.4. Statistical Data Analysis

The evaluation of MLP neural network models was performed using the Statistica 13.3 package (StatSoft, Tulsa, OK, USA) to assess their predictive performance and practical applicability. The coefficient of determination (R^2^) was used to compute the proportion of variance in the experimental data explained by the models. To measure the accuracy of predictions, the mean absolute error (MAE) was applied to determine the average absolute deviation between observed and predicted values (Equation (1)), while the root mean square error (RMSE) provided insight into the goodness of fit between predicted and experimental data (Equation (2)).

(1) $$MAE=\frac{1}{n}\cdot \sum \left|C_{A}-C_{P}\right|$$

(2) $$RMSE=\sqrt{\frac{\sum {\left(C_{A}-C_{P}\right)}^{2}}{n}}$$

where *n* represents the total number of experimental observations and *C_A_* and *C_P_* denote the actual and predicted analyte concentrations, respectively. All statistical analyses and computations were conducted at a significance level of α = 0.05 to ensure the reliability of the evaluation process.

## 3. Results

### 3.1. Dynamics of Thermally Modulated MOS Gas Sensor Responses

As highlighted in the introduction of this paper, extensive research efforts within the volatile compound sector have been directed toward the development of MOS gas sensors that could reliably discriminate various volatile organic compounds [8,9,34,41]. Among them, accurate, rapid, and cost-effective simultaneous identification and quantification of ethanol and methanol remains challenging due to the considerable similarity in their physicochemical properties (such as similar vapor characteristics, low molecular weight, and comparable affinity for sensor surfaces [50]). In the food and pharmaceutical industries, the detection of accidental methanol contamination in alcoholic beverages (which may result from improper fermentation or incomplete separation of methanol during the distillation process) is essential for preventing poisoning, while in sectors such as biofuel, and solvent production, accurately detecting and controlling ethanol and methanol concentrations is crucial for optimizing production processes, ensuring product quality, and maintaining both process efficiency and safety [7,51,52,53]. One recent study attempted the selective detection of specific gases by enhancing the performance of a system (array) consisting of several MOS-based sensors with heterostructures containing gold (Au) decoration. The study also sought to address cross-sensitivity to other gases through machine learning-assisted discriminative analysis; however, the proposed method was limited to the identification and quantification of individual substances [54]. An increase in the selectivity of a MOS gas sensor can also be achieved by utilizing the difference in autoignition temperatures between ethanol (≈369 °C) and methanol (≈433 °C) [55]. This principle was applied in one recent study, in which a MOS gas sensor that demonstrated enhanced selectivity for methanol in methanol–ethanol mixtures when operated at 350 °C was constructed [56].

**Figure 4:**
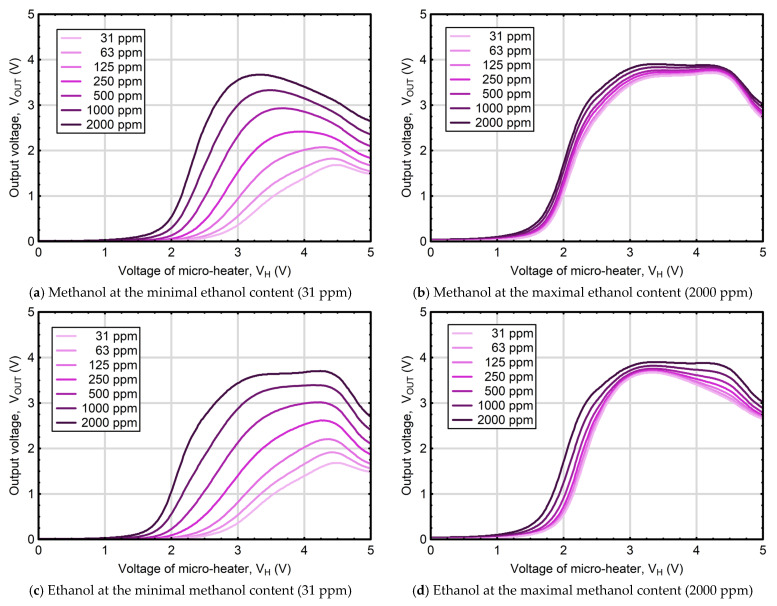
Output voltage responses of the thermally modulated MOS gas sensor for (**a**) varying concentrations of methanol with ethanol fixed at its minimum level (31 ppm), (**b**) varying concentrations of methanol with ethanol at fixed at its maximum level (2000 ppm), (**c**) varying concentrations of ethanol with methanol fixed at its minimum level (31 ppm), and (**d**) varying concentrations of ethanol with methanol fixed at its maximum level (2000 ppm).

As the type of sensors used in the study, equipped with integrated printed RuO_2_ micro-heaters or other Ru-based heaters, typically operate within the ≈200–600 °C range, depending on the architecture of the sensing layer and the micro-heater design [57,58], a promising concept is focused on determining the concentrations of analyzed compounds by differences in the nonlinear behavior of redox processes occurring on its surface as a function of temperature. In this context, a particularly effective approach to differentiating the tested substances is the use of thermally modulated MOS gas sensors, which, by enabling volatile compound analysis under the continuously changing temperature of the sensing layer, produce complex signals resembling fingerprints that encode information related to the composition of the tested atmosphere [59,60]. Figure 4 presents the response patterns of the thermally modulated MOS gas sensor used in this study, recorded in an atmosphere remaining in thermodynamic equilibrium with mixtures containing various ethanol and methanol concentrations.

Comparing the sensor responses for liquid mixtures, in which one accompanying component is kept at a minimal concentration (31 ppm), while the content of the other varies, revealed that the response pattern observed for solutions with increasing methanol concentrations (and minimal ethanol, Figure 4a) differs substantially from the pattern observed for solutions with increasing ethanol concentrations (and minimal methanol, Figure 4c). For mixtures containing a minimal level of the accompanying component (ethanol or methanol), the output voltage (V_OUT_) of the thermally modulated MOS gas sensor remained nearly constant and close to zero within the micro-heater voltage range of 0.0 to 1.0 V. As the micro-heater voltage increased beyond this range, a noticeable rise in V_OUT_ was observed. In the case of samples with maximal methanol content (2000 ppm) and minimal ethanol content, a distinct peak at V_H_ = 3.35 V, corresponding to a maximum recorded output of V_OUT_ = 3.67 V, was registered. In comparison, the sensor response to mixtures with maximal ethanol concentration and minimal ethanol content was also pronounced; however, the V_OUT_ values produced a broader and slightly higher peak within the V_H_ range of 3.25 to 4.3 V, reaching a maximum of V_OUT_ = 3.70 V. It is worth to noting that the waveforms obtained for progressively increasing concentrations of one alcohol, in the presence of a minimal concentration of the second (assisting) component, are well separated and show no overlap across a wide range of heater voltages (V_H_), which greatly facilitates result interpretation. However, at the highest concentrations of the accompanying component (2000 ppm), the response curves become noticeably denser (Figure 4b and 4d), resulting in their overlaps, particularly among those corresponding to lower concentrations of the varying component, making pattern recognition much more challenging, and necessitating the use of advanced approaches capable of identifying even the subtlest differences between waveforms recorded for mixtures with various analyte contents. Taking the above into account, in further considerations the study focused on developing a methodology for quantification of ethanol and methanol in model liquid solutions by integrating the outputs of a thermally modulated MOS gas sensor with PCA–ANN framework data processing.

### 3.2. Reduction in Data Redundancy

In this study, an attempt was made to acquire important information regarding the composition of tested mixtures from the sensor responses using artificial neural networks. However, the direct application of the response patterns from the MOS gas sensor (serving as input data) and the construction of a neural network model for estimating the composition of liquid solutions containing two tested volatile analytes (ANN_E-M_), was hampered by its relatively high dimensionality (each experimental system generated a response vector consisting of 501 points, corresponding to a heater voltage modulated from 0 to 5 V with step of 0.01 V). To ensure the effective use of knowledge contained in the data for ANN_E-M_ model development, it was necessary to reduce the dimensionality of the input vector, while preserving essential information contained in the original dataset. To achieve this, prior to developing the model, PCA, a multivariate unsupervised learning technique, was applied, which has been demonstrated to be effective in extracting essential information from various types of data [61,62,63,64,65,66,67]. Its main advantages include low sensitivity to noise, reduced memory and storage requirements, and improved computational efficiency in subsequent data processing. As PCA is a statistical technique used to analyze phenomena characterized by interrelated variables [68], its use was preceded by the construction of a multivariate correlation matrix to provide an overview of the dependences in the dataset, describing the composition of the tested samples. The obtained correlation matrix revealed strong positive relationships between the variables, with correlation coefficients (R) ranging from 0.586 to 0.999, implying the presence of informational redundancy that confirmed the appropriateness of applying principal component analysis for dimensionality reduction. The conducted analysis allowed us to transform the 501 original correlated variables into 44 new uncorrelated principal components (PCs). Since PCA enhances signal clarity by prioritizing directions of maximum variance, thereby automatically disregarding minor variations often associated with background noise [66,67], the conducted analysis enabled the effective distinction of meaningful data patterns from random fluctuations and the identification of features with significant contributions to the total variance. Based on Kaiser’s and Cattell’s criteria commonly used in PCA [68], the evaluation of the eigenvalues and the proportion of variance explained by each successive principal component led to the selection of first three of them for further analysis. The selected principal components, each with an eigenvalue greater than 1.0, accounted for 99.28% of the total variance in the original dataset and encoded a significant portion of the essential information contained in the waveforms recorded by the thermally modulated MOS gas sensor for mixtures of the target analytes. Hence, in further considerations, the factor values assigned to the selected principal components were subsequently used as input for an artificial neural network to develop a model capable of estimating the composition of liquid solutions based on the gas sensor output data.

### 3.3. Artificial Neural Network Models for Estimating Liquid Mixture Composition

Numerous studies have explored miscellaneous artificial neural network architectures to model biological and chemical systems effectively. Notably, multilayer perceptrons (MLPs) with a single hidden layer have often proven sufficient for solving nonlinear regression problems, aligning with the universal approximation theorem, which states that a feedforward neural network with a single hidden layer and a sigmoidal activation function can approximate any continuous function on a compact domain with arbitrary precision [69]. The usefulness of the mentioned network architecture has been confirmed in numerous studies, including recent works, inter alia, in food and agricultural domains [70,71,72,73].

**Figure 5:**
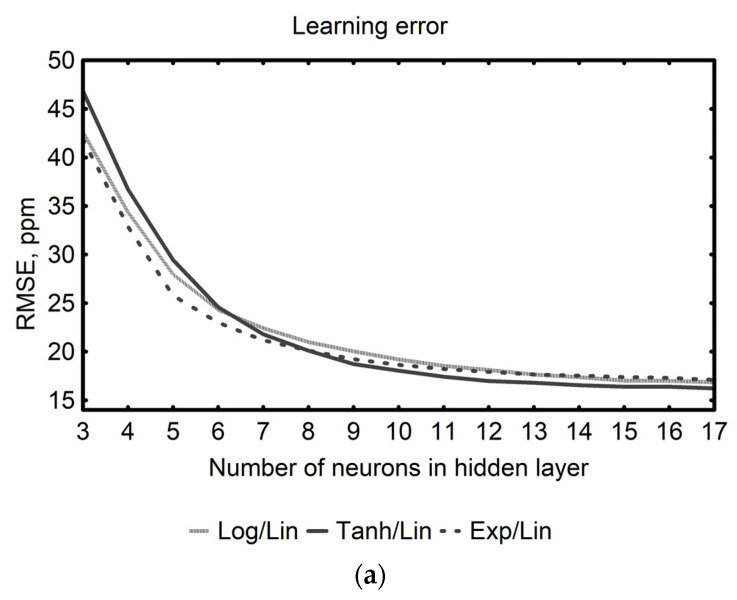

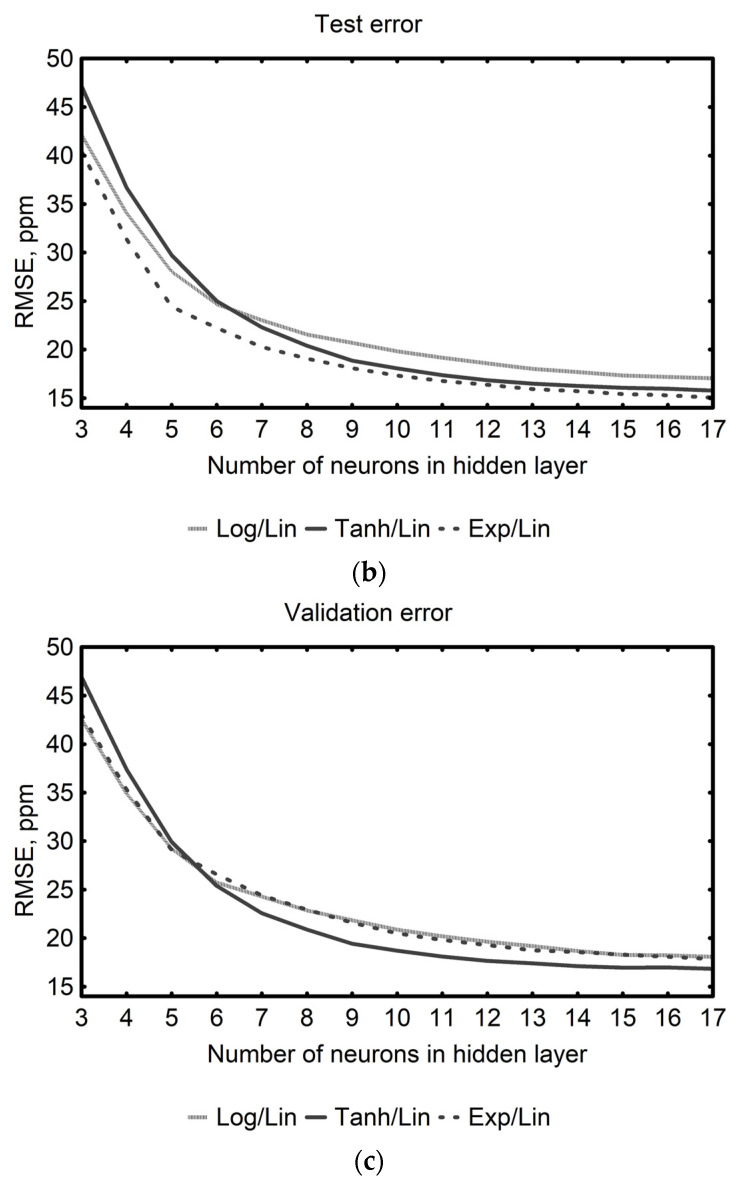
Effect of the number of hidden-layer neurons and the type of activation functions (logistic (Log), hyperbolic tangent (Tanh), and exponential (Exp)) in hidden-layer neurons on the average (**a**) learning, (**b**) test, and (**c**) validation error of MLP neural networks containing a linear function (Lin) in neurons of the output layer applied to estimating the composition of ethanol–methanol liquid mixtures, based on the response patterns of a thermally modulated MOS gas sensor.

In this study, MLPs with a single hidden layer also turned out to be adequate for modeling the composition of liquid mixtures containing two structurally similar volatile components—ethanol and methanol. Systematic optimization of the network structure was conducted by adjusting the number of neurons and the types of activation functions in the hidden layer. Since previous studies have shown that using linear activation functions in neurons of the hidden layer leads to poor performance and limited generalization in regression problems [72,73], only architectures with nonlinear activation functions in neurons of the hidden layer were considered in this study. The comparative assessment of the network architectures revealed a pronounced decrease in both learning and test errors, with an increasing number of neurons in the hidden layer (from three to about eleven) across all examined types of activation functions (Figure 5). Further expansion of the hidden layer size yielded only marginal improvements (e.g., learning error decreased from 18.16 to 16.12 ppm; test error decreased from 18.71 to 15.17 ppm). Considering the type of activation functions used in neurons of the hidden layer, networks with a hyperbolic tangent achieved slightly lower learning errors compared to those employing the logistic and exponential ones. In terms of test error, architectures utilizing the exponential and hyperbolic tangent functions proved somewhat more efficient than those based on the logistic activation function.

The above-presented trend reflects the enhanced capacity of larger networks to model complex nonlinearities; however, it is also well-established that an excessively expanded network may result in overfitting, characterized by the fact that the model memorizes the data on which it was constructed, but performs poorly on new data, thereby reducing its predictive capability [74]. Overfitting can result in deceptively low errors during the learning and testing phases, while significantly impairing generalization, hence, even if learning and test errors provide some indication of model accuracy, relying on them exclusively may yield misleading conclusions. Therefore, to fully assess the model’s quality and ensure that it is not overfitted, in this study the validation error, being the most reliable evaluation metric, was calculated using a separate dataset that did not participate in the learning process. The obtained results (Figure 5) indicated that networks employing logistic and exponential activation functions in the hidden layer consistently exhibited higher validation errors, which, in configurations with more than fourteen hidden neurons, tended to stabilize within the range of 19.75–16.73 ppm. Networks containing hyperbolic tangent function in neurons of the hidden layer offered the best performance. After a significant reduction in the validation error when the hidden layer size was changed from three to eleven neurons, further increasing the number of neurons in the hidden layer resulted in a stabilization of the validation error at around 17.61–16.73 ppm. These results suggest that networks with 11–12 neurons in the hidden layer, equipped with a hyperbolic tangent activation function, might provide a sufficiently structured form to achieve the intended purpose of this study.

Out of the various constructed networks, the one with the lowest weighted average of learning, test, and validation errors was adopted as the model for estimating the composition of ethanol–methanol liquid mixtures (MLP-ANN_E-M_). As expected, the final model selected to describe the compositions of the tested mixtures employed, apart from a linear activation function in the output layer, a hyperbolic tangent activation function in the twelve neurons of the hidden layer. To assess the comprehensiveness and reliability of the elaborated ANN model, basic performance metrics were computed for the entire group of networks with the same architecture (the group of 1000 ANNs generated during the hyperparameter optimization procedure). The average learning, test, and validation errors calculated for all developed networks (Table 1) indicate that the overall performance of the group was satisfactory. Moreover, as each network was created and validated using a different random split of the dataset, the proposed model architecture is relatively insensitive to the specific division of the input data into subgroups. The constructed ANN represents a balanced solution that provides an effective trade-off between topological simplicity and satisfactory performance in predicting the composition of ethanol–methanol liquid mixtures, as it yielded the lowest weighted learning (El), test (Et), and validation (Ev) errors, confirming its efficiency and suitability for practical applications. An overview of the main indicators related to prediction errors and architectural details for the elaborated model is presented in Table 1.

Comparable values of the learning, test, and validation errors of the selected neural network may indicate that the network possesses good generalization ability. The obtained results are consistent with recent studies demonstrating the usefulness of neural network-based architectures for modeling tasks applied to agricultural and biological data [75,76].

#### Evaluation of Model Performance

**Figure 6:**
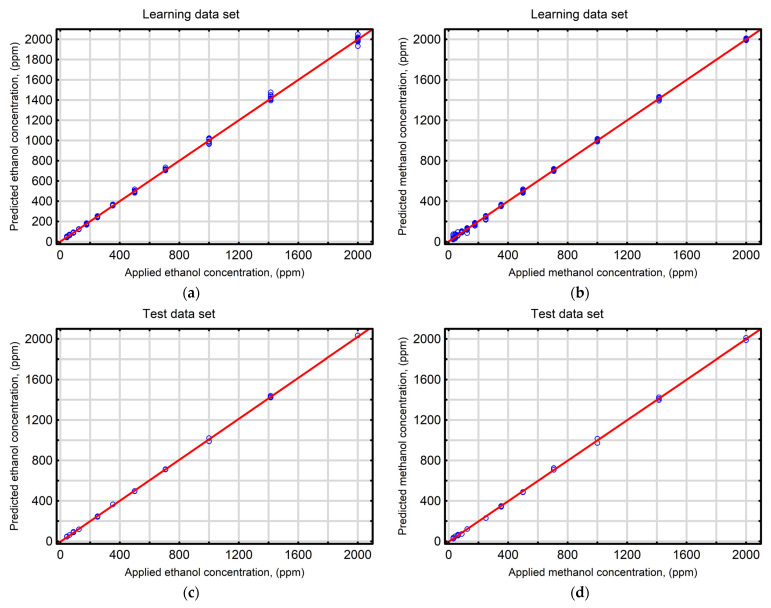

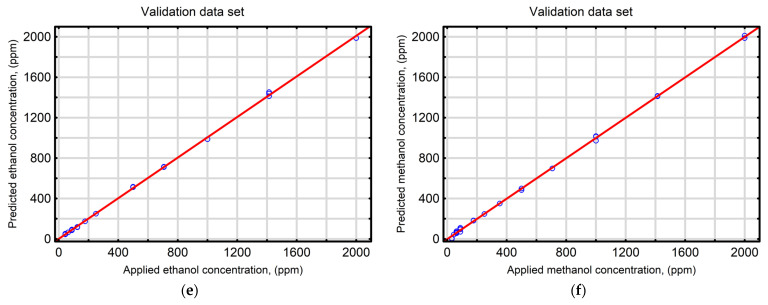
Predicted levels of the tested volatile compounds obtained using the MLP neural network model (MLP-ANNE-M) compared with experimentally applied levels of ethanol for the (**a**) learning, (**c**) test, (**e**) validationdatasets and methanol for the (**b**) learning, (**d**) test, (**f**) validation datasets.

To accurately assess the predictive quality and generalization ability of the network, the developed model was evaluated using suitable statistical indicators recommended for prognostic models. Figure 6, which depicts the comparison between experimental data and predictions from the MLP-ANN_E-M_ model, demonstrates a strong agreement between model responses and analyte concentrations in the tested mixtures in all datasets used for model development and validation (R^2^ = 0.969–0.993). Additionally, low values of the RMSE and MAE presented in Table 2 indicate the high predictive performance of the elaborated model.

The obtained results demonstrate that feature extraction from MOS gas sensor responses using PCA, followed by modeling with traditional ANN based on multilayer perceptrons, provides an effective methodology for both the qualitative and quantitative determination of volatile compounds in mixtures. Thus, it confirmed that PCA in combination with MLPs can serve as a reliable computational tool for processing complex datasets. Previous studies that have employed hybrid approaches integrating B-splines with classical ANNs and CNNs have also demonstrated their high effectiveness in interpreting the output waveforms generated by thermally modulated MOS sensor signals [21,40]. Another recent study proposed the use of an array built on custom-designed gas sensors combined with multiple hybrid machine learning models, including CNNs and ANNs, for the simultaneous detection of volatile organic compounds such as ethanol and methanol and concluded that the type of algorithm employed strongly influences the results, with ensemble-based approaches outperforming traditional or individual deep-learning models [47]. In turn, research by Krivetskiy et al. [43] reported that ANN-based algorithms applied to statistically derived features can markedly improve gas classification accuracy. Collectively, the above-mentioned studies, together with the present one, reinforce the notion that artificial neural networks, regardless of their specific architecture, represent a versatile and powerful tool for enhancing the robustness and selectivity of MOS sensor systems. Nevertheless, it is worth remembering that although effective, the applied CNN-based approach involved complex architectures and additional data interpretation techniques that require substantial computational resources (processor performance, memory capacity). The findings presented in this study represent an extension of previously developed algorithms for the quantitative and qualitative determination of volatile compounds using thermally modulated MOS gas sensors. The proposed methodology employs a single, low-cost, commercially available MOS gas sensor and a simplified neural network model suitable for implementation on low-power microcontroller platforms, providing a more practical and energy-efficient alternative for real-time gas detection. 

Considering the application of the elaborated methodology in real conditions, it should be noted that to ensure the repeatability of results and to validate the core concept of combining the thermal modulation of MOS gas sensors with PCA–ANN data interpretation, experiments were conducted under controlled laboratory conditions. This approach allowed the author to focus on the intrinsic sensor response features associated with different analyte concentrations, independently of external disturbances. It is known that temperature variations can alter baseline resistance and response amplitude, while humidity changes may affect adsorption–desorption dynamics and surface reactions, potentially shifting the PCA feature space and reducing the accuracy of the PCA–ANN model for methanol and ethanol content estimation. Considering the complexity of these processes, for further development of proposed methodology, it will be important to consider how external environmental factors such as ambient temperature and humidity may influence the reliability of the temperature-modulation-based prediction method. Additionally, although Figaro sensors are commonly used and well-validated, future research should include the verification of measurement reproducibility both between sensors of the same type and for repeated use of the same sensor over time, as aging of the sensing layer, exposure to reactive gases, and high temperature may lead to baseline drift and changes in sensitivity, which could affect prediction stability over time. Therefore, to improve its robustness under variable environmental conditions, potential strategies such as incorporating temperature and humidity as auxiliary model inputs for their variability compensation, and the elaboration of adaptive calibration procedures and a model updating approach to compensate for gradual drift should be considered in future studies. Hence, in further research, the author plans to address these aspects to enhance the applicability of the proposed method in real-world scenarios.

## 4. Conclusions

The study confirmed that combining principal component analysis (PCA) with multilayer perceptron (MLP)-based neural networks is an effective strategy for interpreting thermally modulated MOS gas sensor responses and quantifying volatile compounds in liquid mixtures. The PCA step successfully reduced the dimensionality of the recorded signals while maintaining essential features, while the MLP neural networks successfully captured the relationship between the reduced input vector and the corresponding output vector, describing the composition of tested mixtures. The combination of PCA and MLPs therefore provides a reliable computational approach for addressing complex sensor data and enables both qualitative and quantitative assessment of volatile profiles. The results provide strong evidence of the potential of ANN-based methodologies for advancing electronic nose technologies. Future efforts should focus on broadening the range of investigated MOS sensors, addressing more complex gas mixtures and environmental conditions, and integrating adaptive or transfer learning techniques to enhance model generalization. Such developments may pave the way for more accurate, selective, and practical tools for environmental monitoring, food quality assessment, medical diagnostics, and other applications where precise volatile compound detection is essential.

## Figures and Tables

**Table 1:** Summary of the architecture and performance metrics of the following MLP neural networks: the best-performing model for estimating the composition of liquid mixtures containing ethanol and methanol based on the responses of a thermally modulated MOS gas sensor and a group of 1000 networks with the same architecture as the best model. E_l_—learning, E_t_—test, and E_v_—validation errors (all express as the root mean square error, ppm), SD—standard deviation of errors.

Neural Networks Topology	Activation Functions Hidden/Output Layer	Network Group/ Error Metric		Errors (ppm)		
				E_l_	E_t_	E_v_
MLP 3-12-2	Tanh/Lin	Best model		13.27	12.01	12.57
		Group of 1000 networks	mean	16.90	16.68	17.48
		Group of 1000 networks	SD	1.72	2.32	2.52

**Table 2:** Statistical metrics applied to assess the predictive performance of the MLP neural network model for determining the composition of ethanol–methanol liquid mixtures, using data from a thermally modulated MOS gas sensor computed for learning (L), test (T), validation (V), and full (F) datasets.

Statistical Index	Ethanol				Methanol			
	Dataset							
	L	T	V	F	L	T	V	F
Coefficient of determination (R^2^)	0.9994	0.9997	0.9996	0.9995	0.9996	0.9996	0.9995	0.9998
Root mean square error (RMSE)	14.37	11.71	11.28	13.67	11.60	12.30	13.72	12.25
Mean absolute error (MAE)	9.02	8.36	7.38	8.67	8.31	10.02	10.65	8.82

## Data Availability

All data are available from the corresponding author upon request.
